# Mini-Review: Transgenerational CRISPR/Cas9 Gene Editing in Plants

**DOI:** 10.3389/fgeed.2022.825042

**Published:** 2022-02-04

**Authors:** Lennert Impens, Thomas B. Jacobs, Hilde Nelissen, Dirk Inzé, Laurens Pauwels

**Affiliations:** ^1^ Department of Plant Biotechnology and Bioinformatics, Ghent University, Ghent, Belgium; ^2^ VIB Center for Plant Systems Biology, Ghent, Belgium

**Keywords:** CRISPR/Cas9, gene editing, egg cell, pollen, HI-Edit, floral dip

## Abstract

CRISPR/Cas9 genome editing has been used extensively in a wide variety of plant species. Creation of loss-of-function alleles, promoter variants and mutant collections are a few of the many uses of genome editing. In a typical workflow for sexually reproducing species, plants are generated that contain an integrated CRISPR/Cas9 transgene. After editing of the gene of interest, T-DNA null segregants can be identified in the next generation that contain only the desired edit. However, maintained presence of the CRISPR/Cas9 transgene and continued editing in the subsequent generations offer a range of applications for model plants and crops. In this review, we define transgenerational gene editing (TGE) as the continued editing of CRISPR/Cas9 after a genetic cross. We discuss the concept of TGE, summarize the current main applications, and highlight special cases to illustrate the importance of TGE for plant genome editing research and breeding.

## Introduction

CRISPR/Cas9 has rapidly become the predominant tool for plant genome editing (Chen et al., 2019). An important reason is that the CRISPR/Cas9 system only requires co-expression of a generic Cas9 endonuclease and one or more specific single guide RNAs (sgRNA) (Cong et al., 2013). The pairing of the Cas9 ribonucleoprotein complex with target DNA triggers Cas9-mediated DNA cleavage which results in a double stranded break (DSB) (Jinek et al., 2012). The system can easily be engineered to target a DNA region of choice as the specificity is only determined by a ∼20 bp sgRNA spacer complementary to the targeted sequence and a 2–3 bp sequence directly downstream of the target, the protospacer adjacent motif (PAM), which is NGG for *Streptococcus pyogenes* Cas9 (Jinek et al., 2012). DSBs are recognized by endogenous DNA repair mechanisms, of which non-homologous end joining (NHEJ) plays the predominant role in plant cells (Puchta, 2004). When DSBs are repaired perfectly, they are prone to additional rounds of Cas9 cutting. An imperfect repair leads to the creation of an insertion or deletion (indel) at the targeted site, also ending recognition by the sgRNA-guided Cas9 protein. A variety of repair outcomes is possible at each site, although the most often observed edit is a single base pair insertion, often A or T (Bortesi et al., 2016). Alternatively, microhomology-mediated end-joining (MMEJ) may result in larger deletions (>2 bp) through microhomology sites flanking the DSB (van Overbeek et al., 2016). Combinations of insertions and deletions have been reported in *Arabidopsis* through synthesis-dependent MMEJ (Pauwels et al., 2018).

Researchers typically use CRISPR/Cas9 to target exonic open reading frames to generate loss-of-function mutants for functional analysis (Feng et al., 2013; Nekrasov et al., 2013; Fauser et al., 2014; Pauwels et al., 2018). Alternatively, promoter elements or other *cis-*regulatory elements are targeted to disrupt regulation of genes and avoid pleiotropic effects associated with complete loss-of-function (Swinnen et al., 2016)*.* For delivery to plant cells, *Agrobacterium*-mediated transformation is most often used. After transfer of a CRISPR/Cas9 encoding T-DNA molecule to the plant cell, Cas9 and the sgRNA are expressed and are able to edit the target sequence of interest*.* The T-DNA also harbors a selection marker, allowing selection of plants in which the T-DNA has integrated in the genome and is transcriptionally active. The method for stable transformation by *Agrobacterium* differs from species to species. In most plant transformation protocols, explants such as leaves, roots or immature embryos are infected with *Agrobacterium*, after which callus formation is induced in tissue culture. This allows selection of transgenic cells and subsequent regeneration of primary transformants (T0 generation) either by organogenesis or somatic embryogenesis. The model plant *Arabidopsis thaliana* is an exception and allows for the use of *in planta* transformation. In the floral dip method, *Arabidopsis* flowers are brought into contact with *Agrobacterium*, resulting in transformation of haploid female gametophyte cells, before fertilization by self-pollination (Desfeux et al., 2000). Therefore, the primary transformant is the female gametophyte in *Arabidopsis* and the first generation analyzed for gene editing is the T1 generation.

T0 primary transformants in crops, or T1 plants in the case of *Arabidopsis*, are most commonly analyzed by examining the genotype in leaf samples. For diploid plants, often more than two alleles can be found, indicating that the plants are genetic mosaics. These are individuals that have developed from a single cell, and have subsequently acquired mutations during development resulting in the presence of two or more populations of cells with different genotypes (Frank and Chitwood, 2016). Such plants are often referred to as chimeric in the literature, but chimerism denotes the presence of two or more genotypes in a single individual arisen from the conglomeration of cells of more than one genotype in the early stages of development (Frank and Chitwood, 2016). In light of these definitions, the occurrence of multiple different alleles in one plant, caused by incomplete or late CRISPR/Cas9 activity, should be considered mosaicism. Somatic mosaic mutational patterns may indicate that the CRISPR/Cas9 machinery is not always active immediately after *Agrobacterium*-mediated delivery and that different cell lineages already were established. Moreover, leaf samples do not always reveal the genotype of the cells making up the germline and hence the mutations that will be transmitted. For example, a study in *Arabidopsis* found that more than half of mutations in T2 were not present in T1 (Feng et al., 2014).

## Transgenerational Editing

**FIGURE 1:**
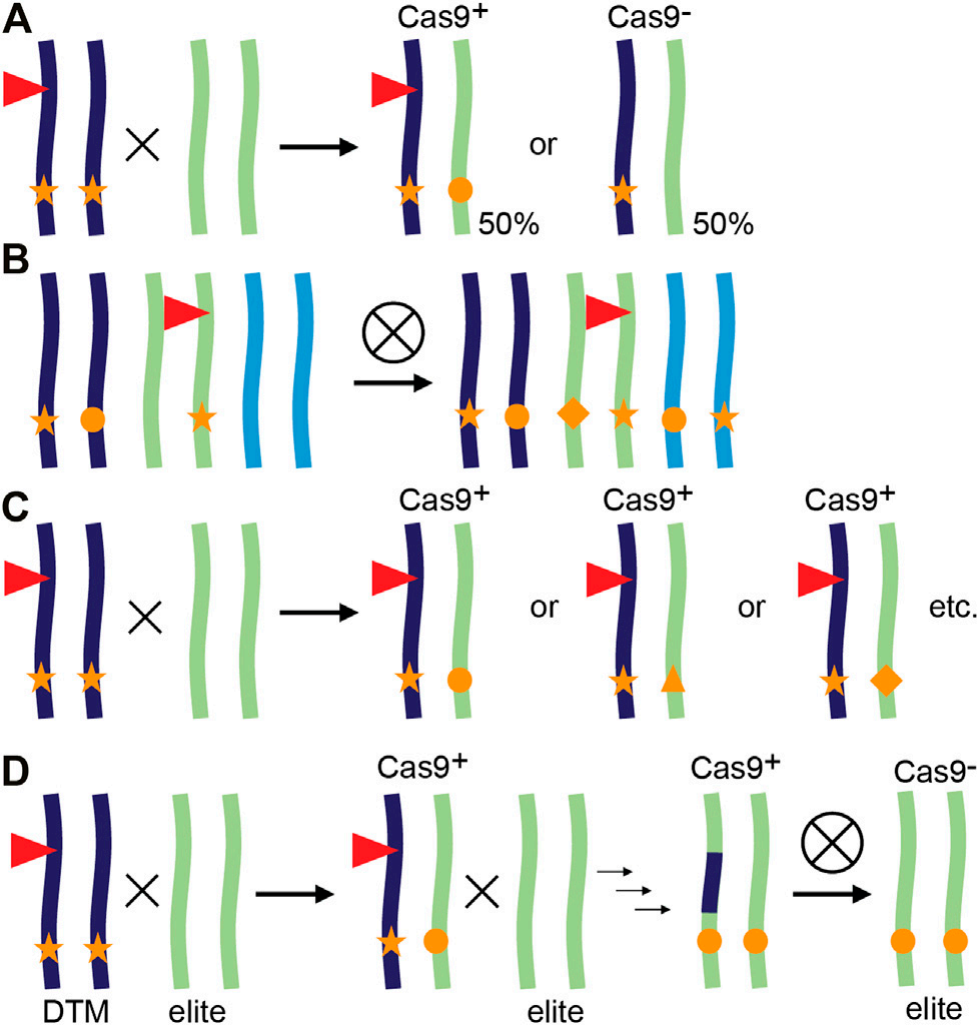
Transgenerational gene editing and applications in plants. **(A)** Principle of transgenerational gene editing (TGE). A transgenic plant represented as a chromosome pair is hemizygous for a CRISPR/Cas9 containing T-DNA locus (red triangle) and edited in both alleles (stars). When crossed with a WT, the resulting progeny either lacks the T-DNA and inherits a single edited allele or inherits the T-DNA, resulting in (transgenerational) editing of the inherited WT allele. **(B–D)** Examples of TGE. **(B)** TGE for continued editing of homoeoalleles in wheat. A transgenic line may have edits only in a subset of homoeoalleles at the homologous chromosomes. After self-crossing and selecting plants that inherited the T-DNA, all homoeoalleles may now be edited (Wang et al., 2018b). **(C)** TGE for allelic variation. In tomato, a loss-of-function mutant (stars) also contains a CRISPR/Cas9 containing T-DNA targeting the promoter of the mutant gene. After a cross with WT, resulting T-DNA containing plants have one loss-of-function allele (star), and an allele with a promoter edit (other symbols). Every individual F1 plant has potentially a different promoter edit and phenotype as the phenotype is not determined by the inherited loss-of-function allele (Rodríguez-Leal et al., 2017). **(D)** Desired-target mutator (DTM) strategy. A maize plant hemizygous for a CRISPR/Cas9 containing T-DNA locus (red triangle) is crossed with an elite inbred line, resulting in TGE and editing of the elite allele. Additional rounds of TGE and backcrossing result in a new edited variety with no linkage drag (Li et al., 2017a).

In a typical workflow for CRISPR/Cas9 gene editing in plants such as maize (*Zea mays*), T0 plants are identified that contain a single CRISPR/Cas9 T-DNA locus and show some degree of editing at the site(s) of interest. After a backcross to wild-type (WT), the T-DNA locus will likely show Mendelian segregation in the progeny and T1 Cas9 null-segregants can be identified. These do not contain the CRISPR/Cas9 transgene but may have inherited a mutant allele from the T0 parent. If so, the mutation is now heterozygous and cannot be mosaic as it went through a single-cell stage, the fertilized egg cell. However, one can also continue with the progeny that still contains a CRISPR/Cas9 transgene. If still active, the Cas9 nuclease will now encounter a novel WT allele introduced by the cross, which can be edited and yield independent alleles (Figure 1A). This continued editing of CRISPR/Cas9 after a genetic cross is referred to as transgenerational gene editing (TGE) (Wang et al., 2018b).

TGE has been used for several applications, although not always named TGE (Figures 1B–D). We provide three examples: editing of additional alleles in polyploid crops, creation of allelic variation and editing of target genes in recalcitrant genetic backgrounds.

### Editing of Homoeoalleles in Polyploid Crops

In the case of polyploid crops such as hexaploid common wheat (*Triticum aestivum*) and tetraploid cotton (*Gossypium hirsutum*), mutations are often only present in a subset of the homoeoalleles targeted by the same sgRNA (Wang et al., 2018a; Wang et al., 2018b; Zhang et al., 2019). Furthermore, efficient transmission and stacking of first-generation mutations becomes increasingly harder, or almost impossible with polyploidy due to Mendelian genetics. By selecting T1 plants that contain the Cas9 transgene, plants can be identified with edits in additional homoeoalleles (Figure 1B, Wang et al., 2018a; Wang et al., 2018b; Zhang et al., 2019). CRISPR/Cas9 can lead to off-target editing when Cas9 makes a DSB at a site with high sequence similarity to the target site and contains a PAM (Hahn and Nekrasov, 2019). Although expressing CRISPR/Cas9 for more than one generation during TGE increases on-target homoeoallele editing, off-targeting is not necessarily increased. Extensive analysis of off-targeting for two sgRNAs in maize T1 plants that contained an active CRISPR/Cas9 module failed to detect any off-targets at sites predicted by genome-wide CIRCLE-seq analysis (Lee et al., 2019). In addition, a multitude of tools are available that allow careful design of spacer sequences to limit sequence similarities, provided a reference genome is available (Haeussler et al., 2016; Hahn and Nekrasov, 2019).

### Creating Novel Genetic Variation

The variety of GE repair outcomes can be exploited to create an array of alleles with potentially different molecular functions, resulting in different phenotypes. As an example, we recently reported independent alleles in the coding region of the maize gene *SAMBA*. Although obtained with the same sgRNA, different phenotypic outcomes were observed and related to translation re-initiation and formation of a truncated protein (Gong et al., 2021). This can be combined with TGE as exemplified in tomato by use of a multiplex mutagenesis drive system to create genetic variation at promoter regions (Figures 1C, Rodríguez-Leal et al., 2017; Wang et al., 2021b). In this system, variation caused by TGE was expanded by combining up to eight sgRNAs targeting the same promoter region (Wang et al., 2021b). Using TGE to create novel genetic variation is also interesting for species or genotypes that are difficult to transform. Obtaining a single CRISPR/Cas9-expressing T0 plant can then be sufficient to create a variety of different alleles in subsequent generations. An example of a difficult-to-transform crop is soybean (*Glycine max*) for which TGE was used to create novel alleles in T1 and T2 generations (Zheng et al., 2020).

### Editing of Recalcitrant Genetic Backgrounds

TGE can also be exploited to introduce mutations in genetic backgrounds that cannot be transformed (Figure 1D). In maize, an *in vivo* desired-target mutator (DTM) strategy was designed to accelerate the breeding process and simultaneously avoid linkage drag compared to introgression of an allele from another variety (Li et al., 2017a). T0 transgenic plants were generated targeting *LIGULELESS1* (*LG1*) and crossed with a WT recalcitrant elite maize inbred line. This resulted in approximately 20% mutation frequency in T1 caused by TGE based on the recessive *lg1* phenotype. One to three additional rounds of TGE and marker assisted backcrossing can subsequently be used to select individuals that are transgene-free and have the desired mutation in the recovered elite background (Li et al., 2017a).

### Combining Haploid Induction and Gene Editing

**FIGURE 2:**
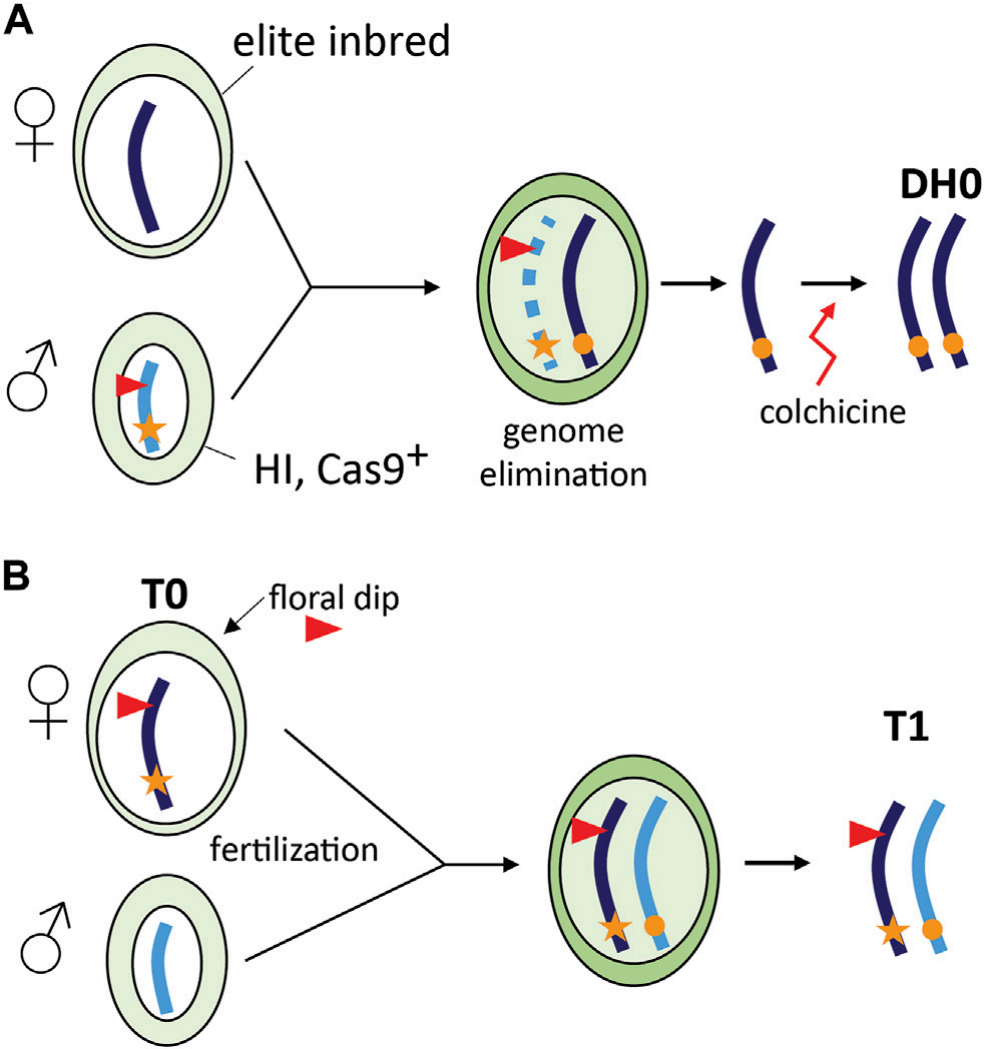
Special cases of TGE. **(A)** Combining haploid induction and gene editing (HI-Edit) in maize. A WT elite maize inbred line is pollinated using a haploid inducer line that contains a CRISPR/Cas9 containing T-DNA locus (red triangle). After fertilization, the male genome is gradually eliminated, but the temporary presence of CRISPR/Cas9 may still edit the elite allele. After doubling of the haploid plant genome using colchicine, a homozygous edited elite DH0 line is obtained (Kelliher et al., 2019; Wang et al., 2019). **(B)** CRISPR/Cas9 gene editing in *Arabidopsis thaliana* using floral dip. The female gametophyte (T0) is transformed using *Agrobacterium tumefaciens* leading to a transformed egg cell that may already be edited. Self-pollination with WT pollen leads to a fertilized egg cell and subsequent zygote in which TGE may take place.

A special case of TGE is the combination of *in vivo* haploid induction and CRISPR/Cas9 gene editing in grasses (Figure 2A). This concept has been first demonstrated in maize and is referred to as haploid inducer (HI)-Edit (Kelliher et al., 2019) or haploid-inducer mediated genome editing (IMGE) (Wang et al., 2019). The technology was developed for editing of elite maize inbreds that are recalcitrant to genetic transformation. In maize, HI lines derived from “stock 6” (Coe, 1959) generate a fraction (∼3%) haploid offspring when used as a pollen donor by an incompletely understood mechanism of paternal genome elimination (Li et al., 2017b). In the HI-Edit strategy, the T-DNA containing the CRISPR/Cas9 construct is transformed or introgressed in a HI line. The resulting line is subsequently used as a pollen donor and crossed with an elite inbred line. As paternal genome elimination likely progresses gradually during the first cell divisions (Jacquier et al., 2020), the temporary expression of the CRISPR/Cas9 machinery from the paternal genome can induce targeted mutations in the remaining maternal genome (Kelliher et al., 2019). Modern maize HI lines produce up to 16% haploids (Kalinowska et al., 2019), while editing of the maternal genome in the maize HI-Edit system currently occurs in only 2–4% of haploids (Kelliher et al., 2019; Wang et al., 2019). As a result, less than 1% of the progeny are edited using this strategy, which underlines the importance of continued research and development in this area. A major difference with TGE in HI-Edit and the other applications is the transient presence of the CRISPR/Cas9 transgene and the resulting transgene-free progeny (Kelliher et al., 2019; Wang et al., 2019; Jacquier et al., 2020).

### CRISPR/Cas9 Editing in *Arabidopsis* Using Floral Dip

In CRISPR/Cas9 editing of *Arabidopsis*, T1 genotypes can be viewed as the result of TGE as a novel WT allele is introduced after transformation of the haploid female gametophyte. Transformation using floral dip results in stable T-DNA insertion in female gametophytes (T0) resulting in seeds (T1) hemizygous for the T-DNA locus (Clough and Bent, 1998; Desfeux et al., 2000). For CRISPR/Cas9-induced mutagenesis, we envisage that if the editing machinery is expressed in the female gametophyte, the haploid cells could already be edited and after fertilization and zygote formation the paternal allele can be edited as well (Figure 2B).

### Role of the Promoter Driving Cas9 in TGE

Early experiments with Cas9 driven by the cauliflower mosaic virus 35S promoter yielded mostly genetic mosaic T1 *Arabidopsis* plants with edits that often could not be inherited (Feng et al., 2014). Hence, edits in the paternal and/or maternal genome were introduced only after the first cell divisions of the early zygote, and additionally not present in the germline. These observations are often attributed to an insufficient activity of the 35S promoter in the germline and very early in development (Kong et al., 2021). Many research groups have investigated the use of alternative promoters to drive Cas9, circumvent mosaicism and achieve germline editing (Wang et al., 2015; Yan et al., 2015). A recent publication describes a strategy for the successful generation of non-mosaic mutants in the T1 generation in *Arabidopsis* (Kong et al., 2021). In this setup, Cas9 is driven by a fusion of the egg cell-specific EC1 promoter with the EC1.2 enhancer and combined with a sgRNA targeting the gene of interest and a sgRNA targeting an endogenous marker gene. Loss-of-function of the latter results in a visual phenotype, on which candidate edited plants are then preselected. It was reported that this system produces plants that are mostly non-mosaic homozygous, transheterozygous (with hetero-allelic mutations) or heterozygous, and only up to 7% mosaic, suggesting that the promoter is highly active in the egg cell, but also in early development stages to allow TGE.

Besides egg cell-specific promoters, pollen-specific promoters have been evaluated for Cas9 expression (Mao et al., 2016; Lei et al., 2020; Jing et al., 2021). In *Arabidopsis*, the promoter of *SPOROCYTELESS,* expressed in sporogenous cells and microsporocytes, was used to drive Cas9. As expected, T1 plants did not show abundant editing while 12–56% of T2 plants showed mutagenesis with up to 88% heterozygous non-mosaic, indicating mutations were inherited from T1-edited germ line cells (Mao et al., 2016). A cross of wheat with maize pollen can result in production of haploid wheat embryos (Laurie and Bennett, 1988) and such an intergeneric wide cross has been used for HI-Edit with CRISPR/Cas9 expressing maize as the pollen donor and a recalcitrant wheat variety as acceptor (Kelliher et al., 2019; Budhagatapalli et al., 2020). In one such use of HI-Edit, the pollen-specific regulatory region of *PROFILIN3* was used to drive Cas9 expression in maize pollen. It was found that several wheat haploids showed large deletions in the target gene (Kelliher et al., 2019). This might imply that also for HI-Edit, cell type-specific expression may be a promising strategy.

## Conclusions and Perspectives

CRISPR/Cas9-based genome editing already is an indispensable tool in plant genetics and breeding and many new technologies are being developed to expand the CRISPR toolbox such as base and prime editing. Many of these new tools could also benefit from TGE-based approaches, especially when editing efficiency is low. A particularly interesting application of TGE is HI-Edit and the research field of haploid induction has seen a number of recent breakthroughs that will impact successful use of HI-Edit in crops. For example, alternative haploid inducers based on CENH3 have now been developed for maize (Wang et al., 2021a) and wheat (Lv et al., 2020). First developed in *Arabidopsis*, haploid inducers based on CENH3 result in maternal genome elimination to produce paternal haploid progeny after pollination with wild-type pollen (Ravi and Chan, 2010). Due to the postzygotic gradual loss of maternal chromosomes, CENH3 systems are compatible with HI-Edit as shown for *Arabidopsis* (Kelliher et al., 2019). A CENH3-based approach in grasses would expand the HI-Edit strategy to both maternal and paternal haploids and may potentially improve overall efficiency of recovering edited plants compared to the stock 6-based system.
